# Immune Infiltration and N(6)-Methyladenosine ncRNA Isoform Detection in Acute Lung Injury

**DOI:** 10.1155/2022/3922299

**Published:** 2022-06-30

**Authors:** Chenzheng Gu, Caiyun Li, Weiwei Wang, Wenhui Yan, Yiwen Yao, Meng Shi, Fei Han, Anquan Shang

**Affiliations:** ^1^Department of Laboratory Medicine, Shanghai Tongji Hospital, School of Medicine, Tongji University, Shanghai 200065, China; ^2^Department of Laboratory Medicine, Pukou Branch of Jiangsu People's Hospital & Nanjing Pukou Dictrict Central Hospital, Nanjing 211800, Jiangsu, China; ^3^Department of Pathology, Tinghu People's Hospital of Yancheng City, Yancheng 224005, Jiangsu, China; ^4^Department of Laboratory Medicine, Yangzhi Rehabilitation Hospital (Shanghai Sunshine Rehabilitation Center), Tongji Univeirsity School of Medicine, Shanghai 201619, China; ^5^Department of Internal Medicine V-Pulmonology, Allergology, Respiratory Intensive Care Medicine, Saarland University Hospital, Homburg 66424, Germany; ^6^Department of Cardiothoracic Surgery, Huashan Hospital, Fudan University, Shanghai 200040, China; ^7^Department of Laboratory Medicine, The Fourth Affiliated Hospital of Nanjing Medical University, Nanjing 210031, Jiangsu, China

## Abstract

Acute lung injury (ALI) is a severe form of sepsis that is associated with a high rate of morbidity and death in critically ill individuals. The emergence of ALI is the result of several factors at work. Case mortality rates might range from 40% to 70%. Researchers have discovered that epigenetic alterations are important in the pathophysiology of ALI and that using epigenetic inhibitors may help reduce symptoms. In embryonic development, circadian rhythm, the cell cycle, and cancer, methylation of m6A seems to be relevant all along the way. According to recent research, posttranscriptional methylation is a key player in the development of alveolar lymphoma. In this study, we clustered ALI based on m6A-related factors, analyzed different classes of immune cell enrichment and inflammatory cytokine expression, screened clustered differential genes for ALI to construct coexpression networks, screened key ALI genes potentially regulated by m6A modifications, and then typed the disease based on key genes to compare the consistency of different clustering results. Our findings have revealed a hitherto undiscovered prognostic sign and a therapeutic target for ALI therapy in m6A and immune invading cells, respectively.

## 1. Introduction

Acute lung injury (ALI) is a severe form of sepsis that is associated with a high rate of morbidity and death in critically ill individuals [1]. Clinically severe hypoxia with diffuse pulmonary infiltrates appeared abruptly in 1967 and were originally defined as acute respiratory distress syndrome and acute lung damage [2]. Alveolar capillary membrane rupture and pulmonary edema are hallmarks of ALI. Furthermore, acute respiratory distress syndrome is a more serious variant of ALI, with a death rate of about 40%. Patients of all ages may be affected by these illnesses, which often occur shortly after a well-detailed trigger event. The emergence of ALI is the result of several factors at work. A person is more prone to suffer from acute lung damage if they have a predisposing condition, such as sepsis, that is severe. Acute lung damage risk varies from patient to patient based on a variety of factors. Examples include drinking, which has been linked to an increased risk of Alzheimer's disease. Direct damage causes alveolar macrophages, T cells, and epithelial cells to produce cytokines and chemokines, increasing lung permeability, causing neutrophil infiltration, and resulting in ALI [3]. When age, underlying chronic diseases, and the degree of non-pulmonary sickness, as well as gas-exchange abnormalities, are taken into consideration along with other risk factors, the results are comparable in both categories of acute lung damage [4].

Acute respiratory distress syndrome is a medical term for when hypoxaemia in acute lung damage is severe (partial arterial oxygen pressure (PaO_2_)/fractional oxygen concentration in inspired air (F_1_O_2_) < 200). Acute lung damage is the general term used in most epidemiological and interventional investigations to describe the larger spectrum of gas-exchange abnormalities (PaO_2_/F_1_O_2_ < 300) [5]. There are limits to these criteria, such as the fact that conventional ventilatory assistance is not required to meet physiological thresholds. It is possible that the use of positive end-expiratory pressure (PEEP) could enhance oxygenation indices to the point that patients who previously met criteria for an acute respiratory distress syndrome will now have acute lung damage, and vice versa. To aid in the clinical diagnosis of ALI, new auxiliary approaches are required.

Changes in gene expression that have nothing to do with the DNA sequence or structure are the focus of epigenetics, a branch of genetics. DNA methylation, histone modification, noncoding RNA, and other epigenetic modifications are examples of these. Atypical epigenetic alteration may be linked to a wide range of disorders, just as a faulty base sequence in traditional genetics. Researchers recently discovered that ALI is caused by numerous epigenetic alterations, and that using epigenetic inhibitors may help to reverse this [6]. Aside from that, noncoding RNA may serve as a biomarker for ALI. Covalent changes to RNA nucleotides are now known to impact RNA stability and translation, allowing them to modulate gene expression. Posttranscriptional regulation occurs when RNA is modified after transcription. There are about 150 different forms of RNA modifications. In addition to messenger and transfer RNA, they are found in ribosomal and small noncoding RNA, as well as in many other forms of RNA (lncRNA). Over 60% of all RNA changes are caused by methylation of RNA [7]. All four ribonucleotides (A, U, C, and G) contain methylation alterations, including N6-methyladenosine (m6A), 5-methylcytosine (m5C), 3-methyluracil (m3U), and N7-methylguanosine (m7G), for example. The most prevalent kind of RNA methylation in mRNA is m6A methylation [8]. The Adenine's sixth methylation site (m6A) is largely found in RRACH RNA sequences, where it regulates splicing, transport, localization, translation, and destruction of target RNAs, among other functions [9]. A widespread lncRNA alteration in higher organisms, m6A is found in a broad range of eukaryotes, including yeast, plants, *Drosophila*, and mammals. According to the research, overexpression of ALKBH5 lowered pre-miR-21 m6A methylation and reduced pre-miR-21 and miR-21-5p levels,. By decreasing the TLR4/MyD88/NF-kB activity in a miR-21-5p-dependent manner, reduction of ALKBH5 protects against radiation-induced lung damage [10]. Even still, little is known about precise molecular roles played by m6A in the immune system's ability to fight infection.

Here, we analyzed the expression of widely reported m6A RNA methylation regulators in ALI using the gene expression omnibus database (GEO database). As a starting point, we looked at the overall pattern of m6A change in the samples from patients with ALI. Two patient groups were derived from a clustering of three m6A gene expression pattern datasets. Later, these genes were utilized to construct a m6A-related gene network, which included weighted gene coexpression network analysis (WGCNA) and a protein-protein interaction (PPI) network. These networks were then built using the genes that had been found to be differentially expressed. The total number of hub genes we discovered was eleven. This hub gene served as a reference for further biological research. In three different m6A-clusters, hub gene expression was organized as follows: cluster2 > cluster1 > cluster3. In the GSE2322 dataset, hub genes were also significantly expressed. Hub gene-cluster subtyping was conducted based on the hub genes to better understand how hub genes work in ALI. After that, we looked at the subtype's correctness in more detail. The Sankey diagram showed that m6A-clusters and hub gene-clusters had a high degree of consistency. M6A-cluster 1 and hub gene-cluster 3 exhibited high levels of consistency, resulting in almost identical expression patterns for hub genes in the two clusters, showing that clustering findings were stable and biologically significant. These findings give a starting point for further research into the connection between m6A and ALI, as well as a solid scientific foundation for treating ALI in the clinic.

## 2. Methods

### 2.1. Data Sources and Preprocessing

All expression microarrays matching ALI keywords were searched in the public database-GEO database. We found GSE10474, GSE2322, GSE3037, and GSE10361 after combing through every publicly available database. There were 91 ALI samples in all. With the help of the *R* package “limma,” we calculated final gene expression levels by multiplying the predicted precision weights of each observation by their corresponding log2. The mean of all probes for a particular gene was calculated. The *R* package “SVA” was used for batch rectification on four data sets to ensure data consistency.

### 2.2. Consensus Clustering of m6A Regulators

All relevant English-language research was thoroughly searched in the PubMed databases from inception to September 28, 2021. Only m6A RNA methylation complex methylation regulator genes found in animals or cells will be considered for this investigation. These genes include METTL3, METTL14, METTL16, WTAP, VIRMA, RBM15, RBM15B, ZC3H13, FTO, ALKBH5, YTHDC1, YTHDC2, IGF2BP1, IGF2BP2, IGF2BP3, YTHDF1, YTHDF2, YTHDF3, HNRNPC, HNRNPA2B1, and RBMX [7, 11–13]. Based on the expression profile of the m6A gene in 91 samples, Pearson correlation of m6A gene expression was calculated and consensus clustering analysis was performed using the *R* package “ConsensusClusterPlus.” Stable clustering results were obtained by 1000 PAM iterations with an 80% sampling ratio in each iteration. The clustering effect was verified by principal component analysis (PCA).

### 2.3. Clustering Expression and Immune Infiltration

The expression of the m6A gene and inflammatory cytokines in different categories were analyzed. The inflammatory cytokines include TNF, IL1B, IL-6, IL8, IL10, IL11, IL15, IL16, and IL18. And ssGSEA enrichment analysis was performed on 28 kinds of immune cells using the *R* package “GSVA.” Using the median value as the threshold, the differences of m6A gene, immune cells, and inflammatory cytokines in different clusters were analyzed by the chi-square test.

### 2.4. Difference Analysis and Weighted Gene Coexpression Network Analysis (WGCNA)

The chi-square test was performed to examine the differential expression of all genes in the expression profile from Step 1 in distinct groups based on clustering findings from Step 2. A difference with a *p*__FDR_ < 0.05 or more was deemed significant. It is becoming more common in bioinformatics to employ WGCNA, a novel systems biology tool, to examine data from gene expression microarray profiling experiments [14]. Modules that are physiologically relevant are discovered once they have been linked to external data. WGCNA may also be utilized to find new treatment targets or biomarkers. Highly linked genes may be organized into modules using a gene coexpression network. By clustering eigengene networks, the first principal component of gene expression for a module—the module eigengene (ME)—was computed as well as the interconnectedness of each module. The module membership (MM) of a gene in the module correlates with its expression profile in both positive and negative ways, with a larger MM number indicating stronger association. As gene significance (GS) increases, so does the biological relevance of a gene's function. GS measures how closely genes are linked to their associated external features. We utilized the WGCNA *R* package to find coexpressed genes in microarrays that had all of their transcripts present. After determining the characteristic, the important modules were determined by looking at how well they are correlated with the clustering category determined in Step 2.

### 2.5. Functional Enrichment Analysis

The *R* package “clusterProfiler” was used for GO and KEGG functional enrichment analysis of all genes in key modules, and *p* < 0.05 was the significance threshold.

### 2.6. Screening for Hub Genes

Genes most related to cluster2 traits (GS > 0.5) and modules (MM > 0.8) in WGCNA key modules were selected as hub genes in modules. All genes in the key module of WGCNA were input into the STRING website (https://string-db.org/) to search for the PPI network, and genes with top 15% (≥6) gene connectivity were selected as the hub genes of the PPI network. WGCNA key genes and PPI key genes were intersected to obtain final hub genes, and the expression of hub genes in different clusters in Step 2 was analyzed.

### 2.7. Analysis of Hub Genes

Based on the expression data of hub genes, the optimal number of classification was determined by finding the optimal SSE inflection point. *K*-means clustering combined with t-SNE dimension reduction was used to type the samples, and the expression of hub genes in different types was further studied. The consistency of m6A gene cluster and the hub genes cluster were analyzed by the Sankey diagram.

### 2.8. Software and Versions

Rx64 3.6.1 was conducted to process data, analyze data, and plot diagrams, and *R* packages included limma, SVA, ConsensusClusterPlus, GSVA, WGCNA, clusterProfiler, and t-SNE. Cytoscape 3.6.1 was performed to plot network diagrams.

## 3. Results

### 3.1. Sixteen m6A RNA Methylation Regulators Were Collected via Systematic Review

**Figure 1:**
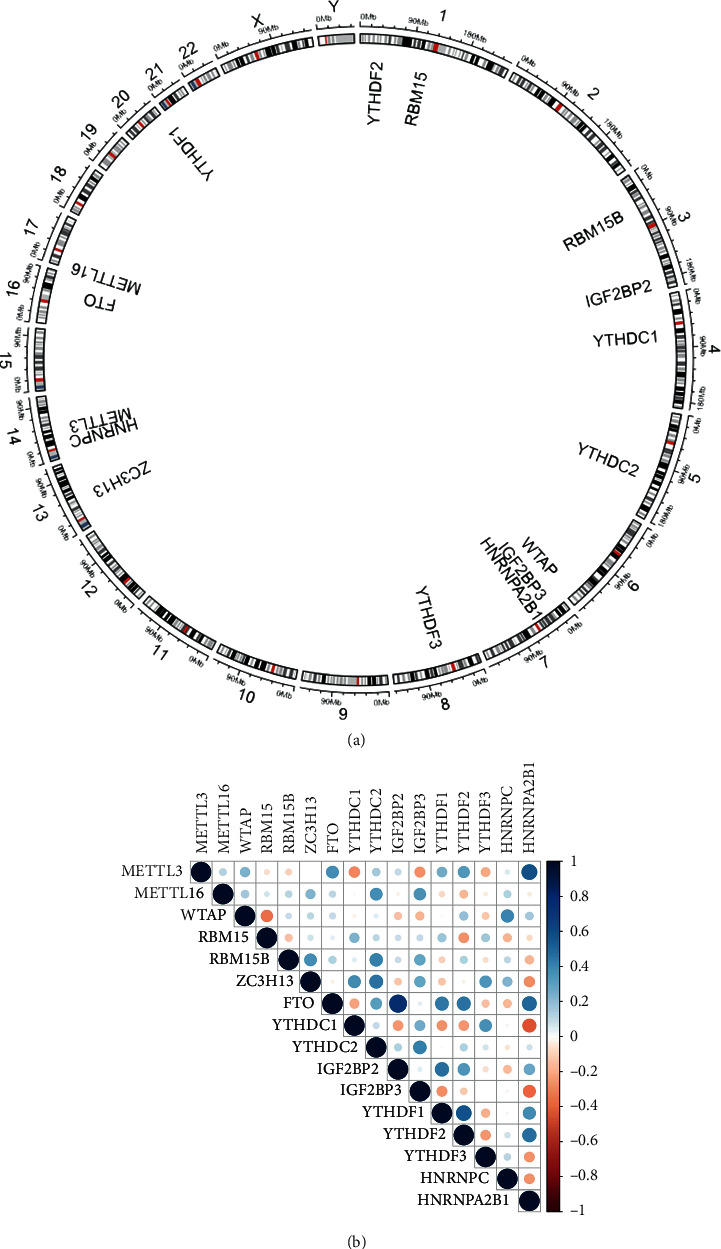
Sixteen m6A RNA methylation regulators were collected. (a) The location of the m6A gene on the chromosome is shown by Circos. (b) The expression correlation of these m6A regulators.

Data preprocessing was used to derive the expression profiles of 12355 genes from 91 samples. We used systematic review to compile a list of 21 m6A regulator genes, then confirmed gene expression using the resulting datasets. Sixteen of the 21 m6A genes were expressed in all four datasets, and the missing genes were METTL14, VIRMA, ALKBH5, IGF2BP1, and RBMX. METTL3, METTL16, WTAP, RBM15, RBM15B, and ZC3H13 have all been identified as m6A writers. YTHDC1, YTHDC2, IGF2BP2, IGF2BP3, YTHDF1, YTHDF2, YTHDF3, HNRNPC, and HNRNPA2B1 were also among m6A readers [15]. Erasers in the m6A family included FTO. These results were shown in Table 1. The chromosomal distribution of 16 m6A regulators was also examined, and these 16 genes were shown to be widely distributed (Figure 1(a)).  Figure 1(b) shows the expression correlation of these m6A genes.

### 3.2. m6A Molecular Subtypes and Consensus Clustering in ALI

**Figure 2:**
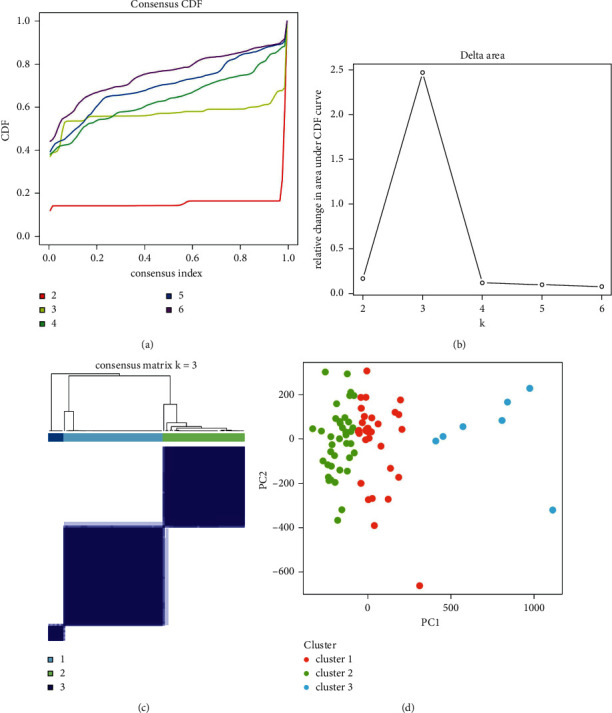
Identification of ALI subtypes based on m6A RNA methylation regulators. (a) CDF curves of consensus scores based on the different subtype number (*k* = 2, 3, 4, 5, 6, 7, 8, 9, and 10) and the corresponding color are represented. (b) The CDF delta area curve of all samples when *k* = 3. (c) Consensus heat maps for *k* = 3. (d) Consensus heat maps for *k* = 3.

**Figure 3:**
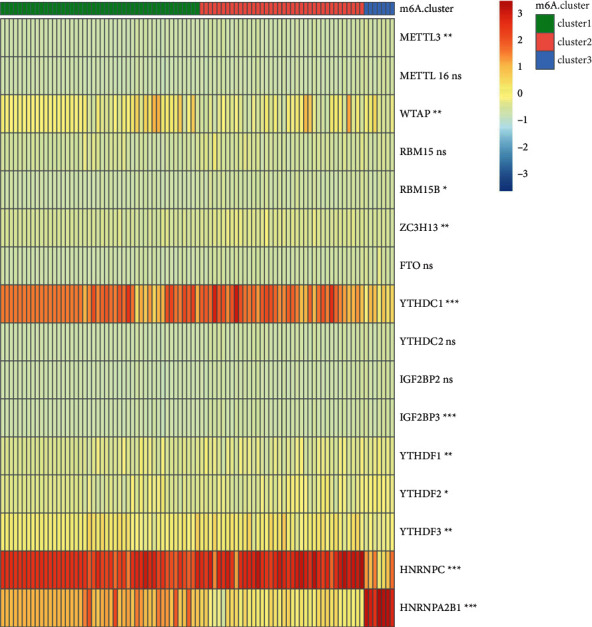
The specific expressions of 16 m6A genes were different in three clusters. Differences of 16 m6A regulators expression levels among different m6A-cluster subtypes. ns, *p* > 0.05; ^*∗*^*p* < 0.05; ^*∗∗*^*p* < 0.01; ^*∗∗∗*^*p* < 0.001; ^*∗∗∗∗*^*p* < 0.0001.

As clustering stability rose from 2 to 6 in cohort, we performed unsupervised cluster analysis on the expression patterns of 16 m6A RNA methylation regulators (Figure 2(a)). It seems that *k* = 3 was a suitable selection with increasing clustering stability in the cohort (Figures 2(b) and 2(c)), including clusters 1, 2, and 3. This was based on the expression similarity of m6A RNA methylation regulators. The sample numbers of each of the three clusters were 46, 38, and 7, respectively. In addition, through PCA verification of the three clusters, the three categories had clear differentiation (Figure 2(d)). As seen in Figure 3, the three clusters had distinct expression patterns for 16 m6A genes.

### 3.3. Immune Status Heterogeneity of m6A-Cluster Subtypes

**Figure 4:**
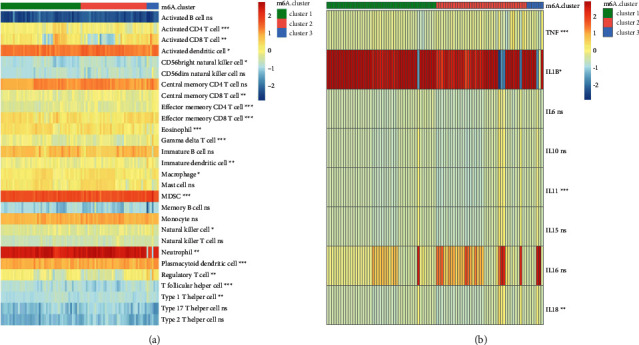
Immune status heterogeneity of m6A-cluster subtypes. (a, b) Differences in immune cell infiltration and cytokines between different m6A-cluster subtypes. ns, *p* > 0.05; ^*∗*^*p* < 0.05; ^*∗∗*^*p* < 0.01; ^*∗∗∗*^*p* < 0.001; ^*∗∗∗∗*^*p* < 0.0001.

A number of different factors may contribute to ALI, including immune cell migration and activation in the lungs, which can lead to damage to the alveolar-capillary membrane. There are many different types of cells involved in this inflammatory process. It is critical to understand ALI's immunological heterogeneity. Immune cells and inflammatory cytokines were evaluated in four different datasets in our research. On the heat map, three distinct groups of immune infiltrating cells could be seen. Activated CD4 and CD8 T cells, as well as neutrophils, and others showed substantial variations across the three immune cell groups (Figure 4(a)).  Figure 4(b) shows the expression levels of three groups of inflammatory cytokine genes. Except for IL8, all four datasets exhibited the same levels of other inflammatory cytokines. Certain cytokines, such as TNF, showed substantial variation across the three groups.

### 3.4. Heterogeneity of Other Biological Function of m6A-Cluster Subtypes

**Figure 5:**
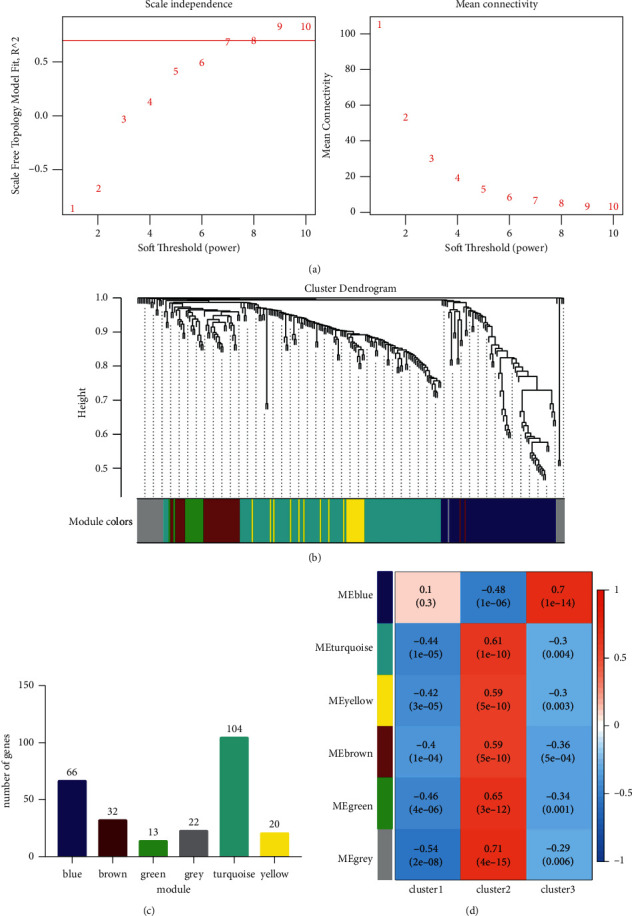
Weighted gene coexpression network analysis. (a) Soft threshold built with the optimum soft value of 8 (*R*^2^ = 0.71). (b) Six modules including, blue, brown, green, grey, turquoise, and yellow, were built. (c) The number of genes in each modules. (d) The relationship between each modules and m6A-cluster (module-trait relationship).

**Figure 6:**
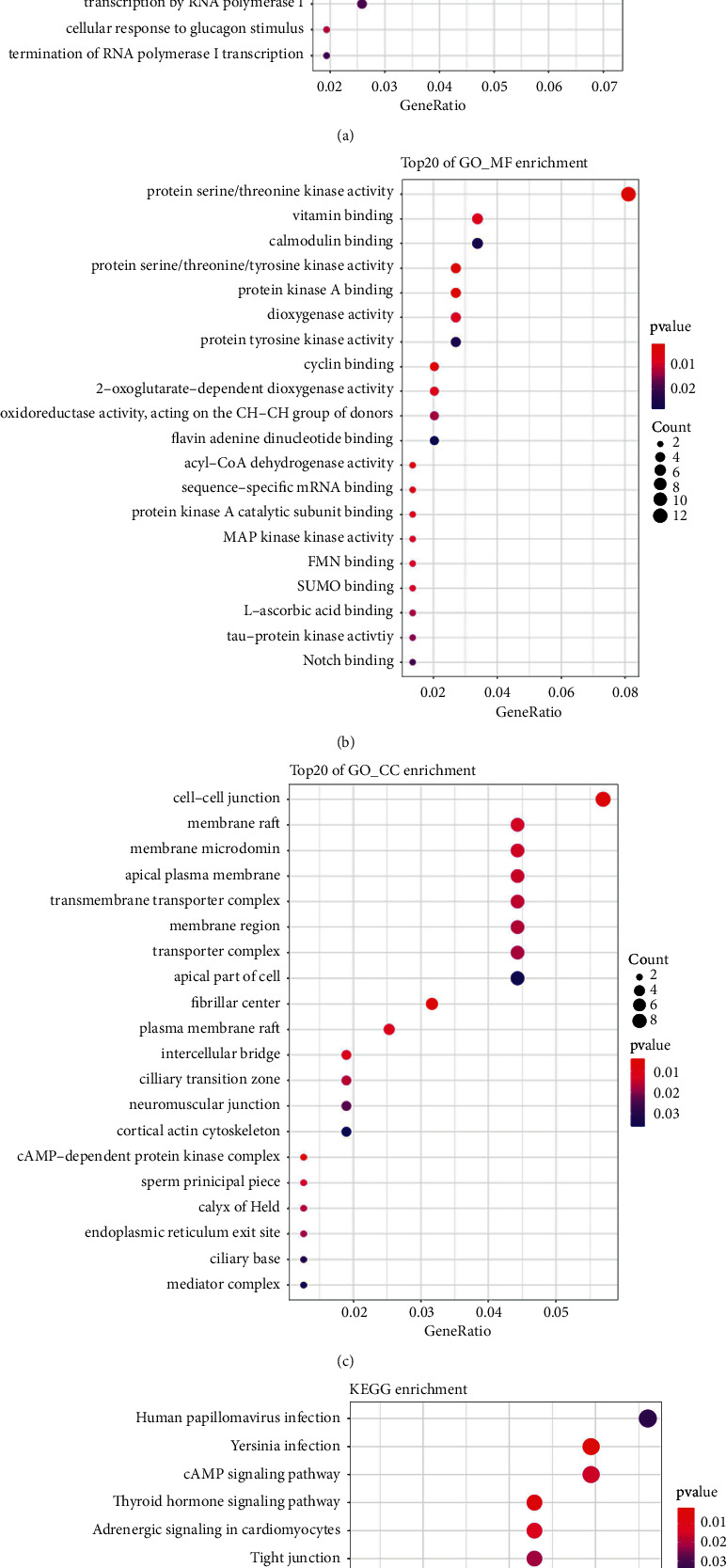
GO and KEGG pathway enrichment of key module genes. (a, b, c) (a), (b), and (c) are the results of GO enrichment analysis at biological progress, molecular function, and cellular component, respectively. (d) KEGG enrichment of key module genes.

The differential expression of 12355 genes in three groups was evaluated after consensus clustering, and 257 genes had substantial changes in their expression. WGCNA analysis was carried out using 257 genes that were found to be different. It was decided to build six modules (Figures 5(b) and 5(c)), each with a soft value of 8 (*R*^2^ = 0.71), to represent the six primary colors of the rainbow (Figure 5(a)). Turquoise, yellow, green, and brown modules were significantly correlated with m6A-cluster (Figure 5(d)), and these four modules were selected as key modules and for further functional analysis. These four key modules contained 169 genes. The results of functional enrichment analysis of 169 genes mainly focused on multicellular organismal homeostasis (Figure 6(a)), protein serine/threonine kinase activity (Figure 6(b)), cell-cell junction (Figure 6(c)), receptor binding, and so on. And KEGG enrichment showed the results mainly focused on human papillomavirus infection, *Yersinia* infection, and so on (Figure 6(d)).

### 3.5. Screening and Analysis of Hub Genes

**Figure 7:**
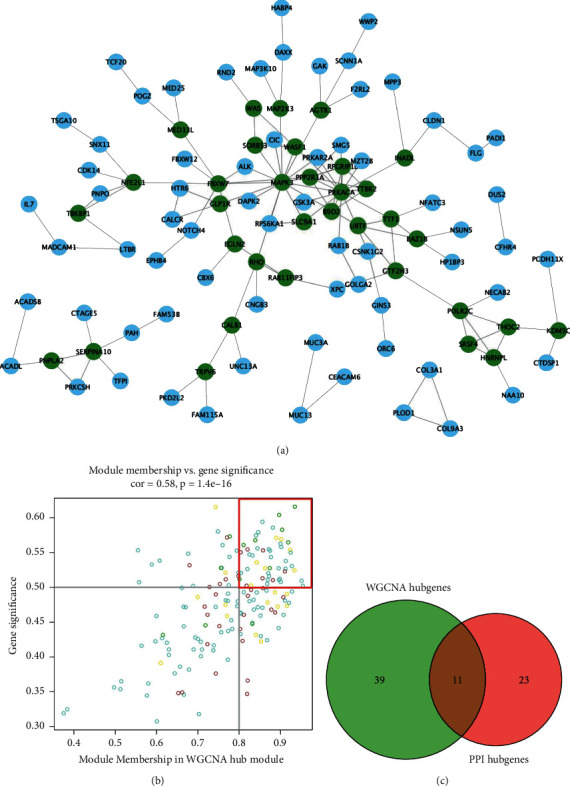
The hub gene network diagram of PPI and WGCNA interactive network screening. (a) The PPI interaction network diagram. (b) Correlation analysis between key modules and gene importance in WGCNA. (c) The PPI network and WGCNA two methods to screen the number of hub genes.

**Figure 8:**
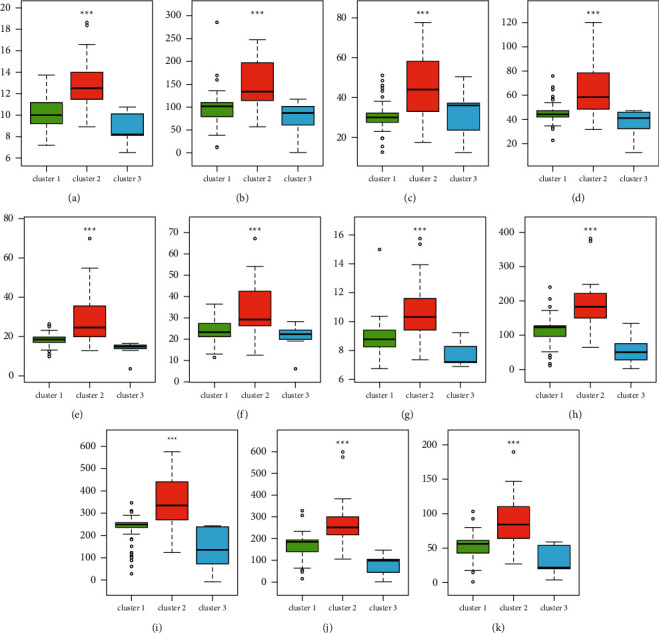
The different expression levels of the hub gene among m6A-cluster subtypes. (a–k) The expression patterns of hub genes in three m6A-clusters including GLP1R, GTF2H3, HNRNPL, POLR2C, RHO, SORBS3, SERPINA10, MAPK3, WAS, EGLN2, and PRKACA. ^*∗∗∗*^*p* < 0.001.

**Figure 9:**
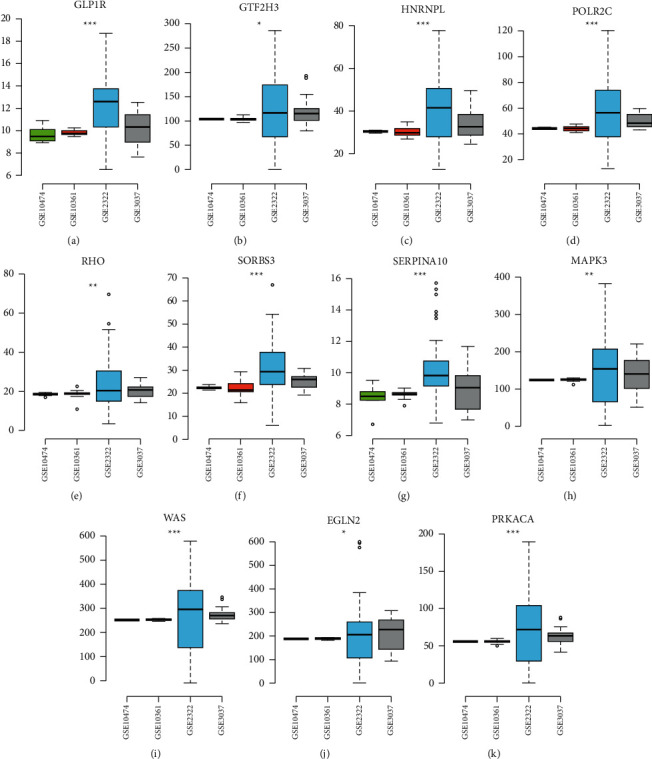
The different expression levels of the hub gene among GEO datasets. (a–k) The expression patterns of hub genes in four GEO datasets. ^*∗*^*p* < 0.05; ^*∗∗*^*p* < 0.01; ^*∗∗∗*^*p* < 0.001.

A total of 169 genes were found in WGCNA important modules, including 50 essential genes (Figure 7(b)). Furthermore, the PPI network revealed the existence of gene correlations in significant modules. The PPI network had 102 genes, with 34 of them being important genes with a link degree ≥3 or higher (shown in green) (Figure 7(a)). We intersected the WGCNA key genes and PPI key genes, and identified 11 hub genes (Figure 7(c)), including GLP1R, GTF2H3, HNRNPL, POLR2C, RHO, SORBS3, SERPINA10, MAPK3, WAS, EGLN2, and PRKACA. Interestingly, the expression patterns of hub genes in three m6A-clusters were cluster2 > cluster1 > cluster3 (Figure 8(a)–8(k)), and hub genes were highly expressed in the GSE2322 dataset (Figure 9(a)–9(k)).

### 3.6. Clustering of Hub Genes and Association Analysis

**Figure 10:**
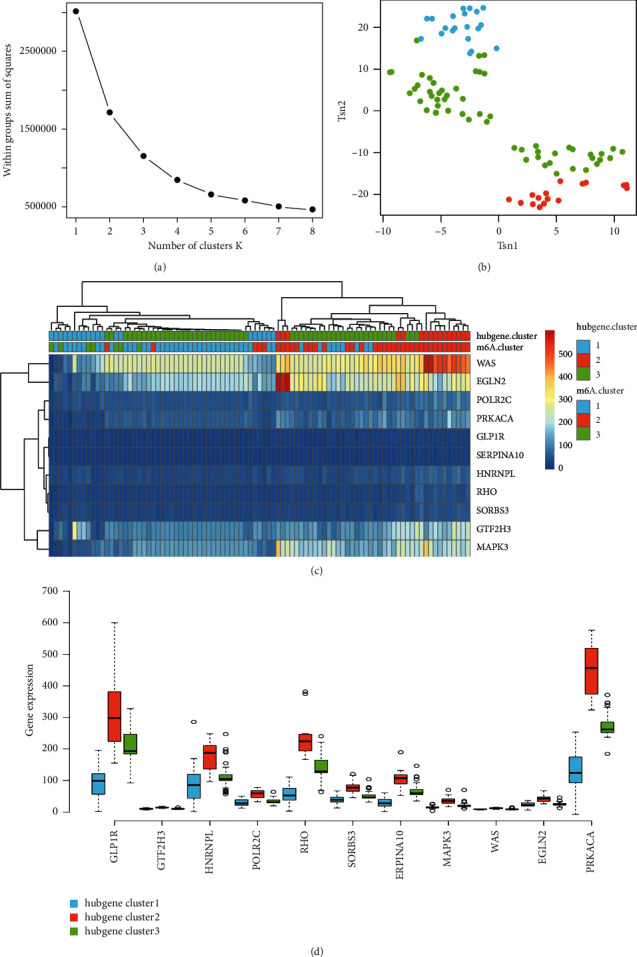
Clustering of hub genes to retype ALI samples. (a) Sum of squares showed samples had the best effect when they were divided into three groups by *K*-means clustering. (b) *t*-SNE dimension reduction analysis. (c) Hub genes expression in m6A-clusters and hub gene-clusters was revealed by the heat map. (d) The expression patterns of hub genes in three hub gene-clusters. ^*∗∗∗*^*p* < 0.001.

**Figure 11:**
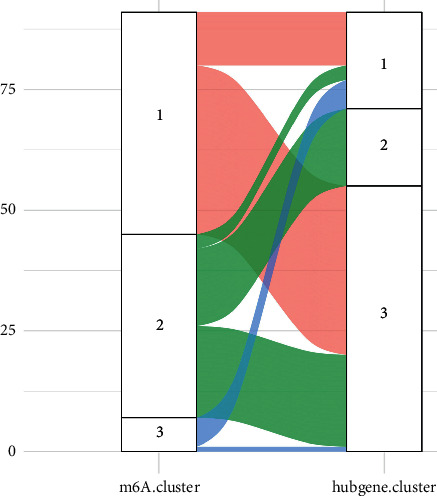
The change from m6A-cluster groups to hub gene-cluster groups. The Sankey diagram showed the change from m6A-cluster groups to cluster gene groups.

Subtypes based on hub genes were created to better understand how ALI's hub genes work. The ideal inflection point approach yielded a total of three kinds (*k* = 3) (Figure 10(a)). The *R* package “t-SNE” dimension reduction analysis revealed the expression patterns of hub genes in the t-SNE1 and t-SNE2 dimensions could substantially differentiate the three groups of samples through sum of squares. And three clusters included cluster 1 (blue) with 20 samples, cluster 2 (red) with 16 samples, and cluster 3 (green) with 55 samples (Figure 10(b)). Hub genes expression in m6A-clusters and hub gene-clusters was revealed by the heat map (Figure 10(c)). Likewise, the expression patterns of hub genes in three hub gene-clusters were cluster2 > cluster3 > cluster1 (Figure 10(d)). Then, we further evaluated the accuracy of the subtype. The Sankey diagram illustrated that there was a high consistency among m6A-clusters and hub gene-clusters (Figure 11).

## 4. Discussion

It is easy to see that acute respiratory distress syndrome (ARDS) and ALI are both well-defined and clearly identifiable clinical illnesses that may be induced by a variety of lung insults or predispositions to lung damage. It is commonly known that this happens a lot in acute care. Treatment for this illness relies heavily on providing great supportive care to critically sick patients, many of whom have comorbid disorders, including sepsis and organ failure. The only known treatment for ALI patients is limited volume mechanical breathing, and fatality rates remain too high to tolerate. A growing corpus of research has recently examined various possible treatment targets for treating ALI, with promising results. The classic TLR4-MYD88 and NFKB signaling pathways play key roles in mTOR-autophagy axis' mediating duties in ALI pathogenesis, with the former serving upstream and the latter serving downstream [16]. Activation of autophagy or mTOR inhibition may be potential therapeutic options to avoid bacterial-induced ALI. As a significant negative autophagy regulator, mTOR has been implicated, but its precise mode of action remains a mystery. Aside from that, it has been shown that METTL3 activated mTORC1 signaling and led to CRC development by inducing GLUT1 translation in a m6A-dependent way [17]. GLUT1, therefore, increased glucose absorption and lactate generation. It was also shown that inhibiting mTORC1 enhanced the anticancer effects of METTL3 silencing in organoids generated from CRC patients as well as METTL3 transgenic mice. As a result of these studies, it was shown that mTOR was crucial in the development of ALI and may have a connection with m6A regulators. Inflammatory cytokines like IL-6 and TNF-*α* are produced in greater amounts when TLRs are activated, which leads to antibacterial responses via downstream signal transduction pathways, particularly activation of nuclear factor *κ*B (NF-*κ*B) and mitogen-activated protein kinase [18]. Infection with SARS-CoV-2, a virus that has recently been linked to ALI and ARDS, has been linked to an inflammatory storm (marked by elevated levels of IL-6, IL-12, and IL-1, as well as TNF and deficient type I interferon activity) [19]. As with patients who have received CAR T cells treatment or antibodies that engage bispecific T cells, this inflammatory reaction is similar to cytokine release syndrome that may be addressed with anti-cytokine therapy that targets the IL-6–IL-6 receptor (IL-6R) signaling pathway [20]. HESH3-lysine-27 trimethylation (H3K27me3) was shown to induce demethylation of histone H3, leading to an increase in the production of proinflammatory cytokines and making m6A more readily available for the cotranscriptional storage [6]. ALI is characterized by excessive intra-alveolar fibrin deposition, driven, at least in part by inflammation. The imbalance between activation of coagulation and inhibition of fibrinolysis in patients with ALI favors fibrin formation and appears to occur both systemically and in the lung and airspace [21]. Intravascular coagulation factor predominates over the anticoagulant factor at ALI, breaking the equilibrium state of maintaining blood mobility. Another study revealed that activated platelets induce neutrophil extracellular traps (NETs) formation, and NETs can increase the permeability of endothelial monolayers, and NETs were found in the lungs in both experimental and clinical transfusion-related acute lung injury (TRALI) [22]. Preventing platelet activation or interfering with NET constituents results in marked lung protection in experimental TRALI, suggesting that NETs may serve as a novel therapeutic target to treat patients suffering from this severe condition.

**Figure 12:**
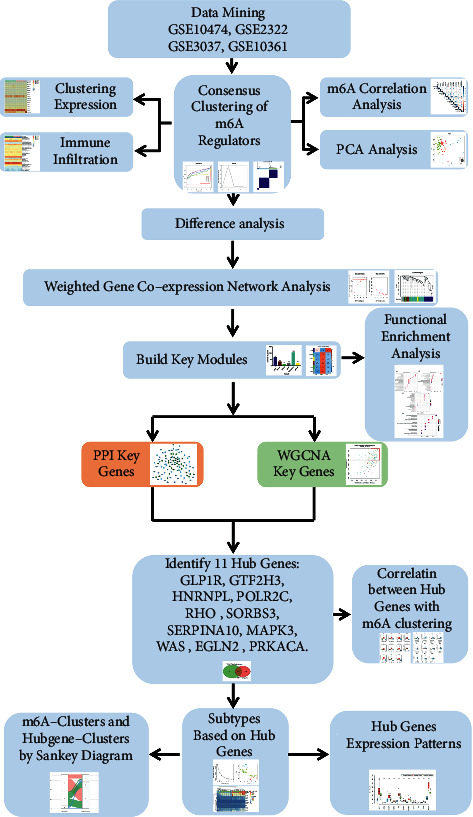
Graphical abstract: A flow chart of the study.

A search of all expression microarrays matching ALI keywords in the public database-GEO database was performed to get a better understanding of the relationship between m6A methylation and ALI. And we draw a graphical abstract to demonstrate the analysis workflows of this study (Figure 12). We collected GSE10474, GSE2322, GSE3037, and GSE10361 by removing unnecessary information. We collected the total of 91 ALI samples with 12355 genes. The ALI samples were retyped into three groups using consensus clustering, and each of the three clusters could be distinguished from the others. A comprehensive study yielded a list of 16 m6A regulator genes. METTL3, METTL16, WTAP, RBM15, RBM15B, and ZC3H13 have all been identified as m6A writers. YTHDC1, YTHDC2, IGF2BP2, IGF2BP3, YTHDF1, YTHDF2, YTHDF3, HNRNPC, and HNRNPA2B1 were also among m6A readers. Erasers in the m6A family included the FTO.

After that, we checked the datasets for evidence of gene expression. There are many different types of cells involved in this inflammatory process. Clarification of ALI's immune state heterogeneity is required. To find out more about immune cells and inflammatory cytokines, we looked at four different datasets. On the heat map, three distinct groups of immune infiltrating cells could be seen. Macrophages differed significantly from neutrophils and TNF-*α* from activated CD4 T cells in three subtypes. Resident and recruited macrophages have been shown to play an important role in the initiation and maintenance of pulmonary inflammation after a lung infection or damage. TNF-*α* is highly secreted by alveolar macrophages, but interstitial macrophages are better at releasing interleukin-1*β* (IL-1*β*) and IL-6. The next step, we intersected the WGCNA key genes and PPI key genes, and identified 11 hub genes, including GLP1R, GTF2H3, HNRNPL, POLR2C, RHO, SORBS3, SERPINA10, MAPK3, WAS, EGLN2, and PRKACA, and analyzed the expression patterns of hub genes (cluster2 > cluster1 > cluster3). The overview of all hub genes, including the function and introduction, was shown in Table 2. The hub gene-cluster subtype was performed based on hub genes to better understand how hub genes operate in ALI. The consistency and accuracy of these subtypes were then assessed. There were 35 shared samples between m6A-cluster 1 and hub gene-cluster 3, with a share of shared samples in subtype clusters of 76% and 64%, respectively. Each cluster had 16 common samples, which accounted for 42% of the total and 100% of the hub gene-cluster 2 samples. Figures 8 and 10(d) show that hub genes were found to be widely expressed, with the greatest levels of expression occurring in cluster 2 of various subtypes (Table 3). M6A-cluster 1 and hub gene-cluster 3 exhibited high levels of consistency, resulting in almost identical expression patterns for hub genes in the two clusters, showing that clustering findings were stable and biologically significant.

## 5. Conclusions

ALI is a common disease in adult and pediatric intensive care units with high morbidity and mortality. m6A modifications play important roles in mammals, such as upregulation of RNA stability, localization, transport, shearing, and translation at the posttranscriptional level, and play an important role in the development of the disease. The pathophysiology of ALI originates from a severe inflammatory response. In this study, we clustered ALI based on m6A-related factors, analyzed different classes of immune cell enrichment and inflammatory cytokine expression, screened clustered differential genes for ALI to construct coexpression networks, screened key ALI genes potentially regulated by m6A modifications, and then typed the disease based on key genes to compare the consistency of different clustering results. Our findings have revealed a hitherto undiscovered prognostic sign and therapeutic target for ALI therapy in m6A and immune invading cells, respectively.

## Figures and Tables

**Table 1:** The name and type of m6A-related genes.

Gene symbol	Types
METTL3	m6A writers
METTL16	m6A writers
WTAP	m6A writers
RBM15	m6A writers
RBM15B	m6A writers
ZC3H13	m6A writers
YTHDC1	m6A readers
YTHDC2	m6A readers
IGF2BP2	m6A readers
IGF2BP3	m6A readers
YTHDF1	m6A readers
YTHDF2	m6A readers
YTHDF3	m6A readers
HNRNPC	m6A readers
HNRNPA2B	m6A readers
FTO	m6A erasers

**Table 2:** The function of 11 hub genes.

Gene symbol	Abbreviations	Function
Glucagon-like peptide 1 receptor	GLP1R	This gene encodes a 7-transmembrane protein that functions as a receptor for glucagon-like peptide 1 (GLP-1) hormone, which stimulates glucose-induced insulin secretion [23].
General transcription factor IIH subunit 3	GTF2H3	This gene encodes a member of the TFB4 family. The encoded protein is a subunit of the core-TFIIH basal transcription factor and localizes to the nucleus [24].
Heterogeneous nuclear ribonucleoprotein L	HNRNPL	Functional annotation defined a set of essential spliceosome and RNA binding protein (RBP) genes [25].
RNA polymerase II subunit C	POLR2C	This gene encodes the polymerase responsible for synthesizing messenger RNA in eukaryotes [26].
Rhodopsin	RHO	Protein encoded by this gene is found in rod cells in the back of the eye and is essential for vision in low-light conditions [27].
Sorbin and SH3 domain containing 3	SORBS3	SORBS3gene codes for the adapter protein vinexin, and has been shown to play a role in growth-factor-induced signal transduction and a cytoskeleton structure [28].
Serpin family a member 10	SERPINA10	Protein encoded by this gene belongs to the serpin family, predominantly expressed in the liver and secreted in plasma. It inhibits the activity of coagulation factors Xa and XIa in the presence of protein *Z*, calcium, and phospholipid [29].
Mitogen-activated protein kinase 3	MAPK3	Protein encoded by this gene is a member of the MAP kinase family. MAP kinases, act in a signaling cascade that regulates various cellular processes such as proliferation, differentiation, and cell cycle progression in response to a variety of extracellular signals [30].
WASP actin nucleation promoting factor	WAS	In addition to its role in the cytoplasmic cytoskeleton, also promotes actin polymerization in the nucleus, thereby regulating gene transcription and repair of damaged DNA [31].
EGL-9 family hypoxia inducible factor 2	EGLN2	EGLN2 is involved in regulating hypoxia tolerance and apoptosis in cardiac and skeletal muscle [32].
Protein kinase CAMP-activated catalytic subunit alpha	PRKACA	In hepatocellular fibrolamellar carcinoma (FL-HCC), the fusion DNAJB1 to PRKACA is suggested to be a diagnostic marker for this rare subtype of HCC [33].

**Table 3:** Comparison of two clustering results.

	m6A-cluster	Hub gene-cluster
cluster1	46	20
cluster2	38	16
cluster3	7	55

## Data Availability

The data supporting the findings of this study are available from the corresponding author upon request.

## Abbreviations

ALI: Acute lung injury
m6A: N6-methyladenosine
m5C: 5-Methylcytosine
m3U: 3-Methyluracil
m7G: N7-methylguanosine
GEO database: Gene expression omnibus database
PPI: Protein-protein interaction network
WGCNA: Weighted gene coexpression network analysis
ME: Module eigengene
MM: Module membership
GS: Gene significance
ARDS: Acute respiratory distress syndrome
NETs: Neutrophil extracellular traps.

## References

[1] Zarrin A. A., Bao K., Lupardus P., Vucic D. (2021). Kinase inhibition in autoimmunity and inflammation. *Nature Reviews Drug Discovery*.

[2] Ashbaugh D. G., Boyd Bigelow D., Petty T. L., Levine B. E. (1967). Acute respiratory distress in adults. *The Lancet*.

[3] Atkin-Smith G. K., Duan M., Chen W., Poon I. K. H. (2018). The induction and consequences of Influenza A virus-induced cell death. *Cell Death & Disease*.

[4] Zilberberg M. D., Epstein S. K. (1998). Acute lung injury in the medical ICU: comorbid conditions, age, etiology, and hospital outcome. *American Journal of Respiratory and Critical Care Medicine*.

[5] Wheeler A. P., Bernard G. R. (2007). Acute lung injury and the acute respiratory distress syndrome: a clinical review. *The Lancet*.

[6] Wu C., Chen W., He J. (2020). Interplay of m(6)A and H3K27 trimethylation restrains inflammation during bacterial infection. *Science Advances*.

[7] Meyer K. D., Jaffrey S. R. (2017). Rethinking m(6)A readers, writers, and erasers. *Annual Review of Cell and Developmental Biology*.

[8] Malbec L., Zhang T., Chen Y. S. (2019). Dynamic methylome of internal mRNA N(7)-methylguanosine and its regulatory role in translation. *Cell Research*.

[9] Roundtree I. A., Evans M. E., Pan T., He C. (2017). Dynamic RNA modifications in gene expression regulation. *Cell*.

[10] Liu Z., Li R., Wang Y. (2021). *ALKBH5 Regulates m6A Methylation-Modi Ed Pre- miR-21 to Promote Lung Injury in Obstructive Sleep Apnea Rat Model*.

[11] Reichel M., Köster T., Staiger D. (2019). Marking RNA: m6A writers, readers, and functions in arabidopsis. *Journal of Molecular Cell Biology*.

[12] Zhang C., Fu J., Zhou Y. (2019). A review in research progress concerning m6A methylation and immunoregulation. *Frontiers in Immunology*.

[13] Vu L. P., Pickering B. F., Cheng Y. (2017). The N(6)-methyladenosine (m(6)A)-forming enzyme METTL3 controls myeloid differentiation of normal hematopoietic and leukemia cells. *Nature Medicine*.

[14] Langfelder P., Horvath S. (2008). WGCNA: an *R* package for weighted correlation network analysis. *BMC Bioinformatics*.

[15] Xu X., Huang J., Ocansey D. K. W. (2021). The emerging clinical application of m6A RNA modification in inflammatory bowel disease and its associated colorectal cancer. *Journal of Inflammation Research*.

[16] Hu Y., Lou J., Mao Y. Y. (2016). Activation of MTOR in pulmonary epithelium promotes LPS-induced acute lung injury. *Autophagy*.

[17] Chen H., Gao S., Liu W. (2021). RNA N(6)-methyladenosine methyltransferase METTL3 facilitates colorectal cancer by activating the m(6)A-GLUT1-mTORC1 *Axis* and is a therapeutic target. *Gastroenterology*.

[18] Moon M. K., Cho B. J., Lee Y. J. (2012). The effects of chronic exercise on the inflammatory cytokines interleukin-6 and tumor necrosis factor-*α* are different with age. *Applied Physiology Nutrition and Metabolism*.

[19] Ren L. L., Wang Y. M., Wu Z. Q. (2020). Identification of a novel coronavirus causing severe pneumonia in human: a descriptive study. *Chinese Medical Journal*.

[20] He H., Liao Q., Zhao C. (2021). Conditioned CAR-T cells by hypoxia-inducible transcription amplification (HiTA) system significantly enhances systemic safety and retains antitumor efficacy. *Journal for immunotherapy of cancer*.

[21] Sebag S. C., Bastarache J. A., Ware L. B. (2011). Therapeutic modulation of coagulation and fibrinolysis in acute lung injury and the acute respiratory distress syndrome. *Current Pharmaceutical Biotechnology*.

[22] Caudrillier A., Kessenbrock K., Gilliss B. M. (2012). Platelets induce neutrophil extracellular traps in transfusion-related acute lung injury. *Journal of Clinical Investigation*.

[23] Lim G. B. (2019). GLP1R agonists: primary cardiovascular prevention and oral administration. *Nature Reviews Cardiology*.

[24] Rahbar K., Afshar-Oromieh A., Seifert R. (2018). Diagnostic performance of (18)F-PSMA-1007 PET/CT in patients with biochemical recurrent prostate cancer. *European Journal of Nuclear Medicine and Molecular Imaging*.

[25] Fei T., Chen Y., Xiao T. (2017). Genome-wide CRISPR screen identifies HNRNPL as a prostate cancer dependency regulating RNA splicing. *Proceedings of the National Academy of Sciences of the USA*.

[26] Moriwaki M., Moore B., Mosbruger T. (2017). POLR2C mutations are associated with primary ovarian insufficiency in women. *Journal of the Endocrine Society*.

[27] Rao V. R., Cohen G. B., Oprian D. D. (1994). Rhodopsin mutation G90D and a molecular mechanism for congenital night blindness. *Nature*.

[28] Kioka N., Ueda K., Amachi T. (2002). Vinexin, CAP/ponsin, ArgBP2: a novel adaptor protein family regulating cytoskeletal organization and signal transduction. *Cell Structure and Function*.

[29] Han X., Fiehler R., Broze G. J. (1998). Isolation of a protein Z-dependent plasma protease inhibitor. *Proceedings of the National Academy of Sciences*.

[30] Fan H. Y., Liu Z., Shimada M. (2009). MAPK3/1 (ERK1/2) in ovarian granulosa cells are essential for female fertility. *Science*.

[31] Taylor M. D., Sadhukhan S., Kottangada P. (2010). Nuclear role of WASp in the pathogenesis of dysregulated TH1 immunity in human wiskott-aldrich syndrome. *Science Translational Medicine*.

[32] Epstein A. C., Gleadle J. M., McNeill L. A. (2001). *C. elegans* EGL-9 and mammalian homologs define a family of dioxygenases that regulate HIF by prolyl hydroxylation. *Cell*.

[33] Honeyman J. N., Simon E. P., Robine N. (2014). Detection of a recurrent DNAJB1-PRKACA chimeric transcript in fibrolamellar hepatocellular carcinoma. *Science*.

